# Applications of Tandem Mass Spectrometry (MS/MS) in Protein Analysis for Biomedical Research

**DOI:** 10.3390/molecules27082411

**Published:** 2022-04-08

**Authors:** Anca-Narcisa Neagu, Madhuri Jayathirtha, Emma Baxter, Mary Donnelly, Brindusa Alina Petre, Costel C. Darie

**Affiliations:** 1 Laboratory of Animal Histology, Faculty of Biology, “Alexandru Ioan Cuza” University of Iasi, Carol I bvd. No. 22, 700505 Iasi, Romania; 2 Biochemistry & Proteomics Group, Department of Chemistry and Biomolecular Science, Clarkson University, 8 Clarkson Avenue, Potsdam, NY 13699, USA; jayathm@clarkson.edu (M.J.); baxterem@clarkson.edu (E.B.); donnelm@clarkson.edu (M.D.); brindusa.petre@uaic.ro (B.A.P.); 3 Laboratory of Biochemistry, Faculty of Chemistry, “Alexandru Ioan Cuza” University of Iasi, Carol I bvd. No. 22A, 700505 Iasi, Romania

**Keywords:** proteomics, tandem mass spectrometry (MS/MS), biomedical research, biomarkers

## Abstract

Mass Spectrometry (MS) allows the analysis of proteins and peptides through a variety of methods, such as Electrospray Ionization-Mass Spectrometry (ESI-MS) or Matrix-Assisted Laser Desorption Ionization-Mass Spectrometry (MALDI-MS). These methods allow identification of the mass of a protein or a peptide as intact molecules or the identification of a protein through peptide-mass fingerprinting generated upon enzymatic digestion. Tandem mass spectrometry (MS/MS) allows the fragmentation of proteins and peptides to determine the amino acid sequence of proteins (top-down and middle-down proteomics) and peptides (bottom-up proteomics). Furthermore, tandem mass spectrometry also allows the identification of post-translational modifications (PTMs) of proteins and peptides. Here, we discuss the application of MS/MS in biomedical research, indicating specific examples for the identification of proteins or peptides and their PTMs as relevant biomarkers for diagnostic and therapy.

## 1. Introduction

Going through the current era of predictive, preventive, and personalized “3P” medicine [1] accompanied by a wide “omics revolution” [2], proteomics techniques are largely applied in biological and both fundamental and clinical research. Proteomics approaches have been employed in the last few decades for detecting and discriminating the early stages of diseases and for precise diagnoses to allow quick medical decisions and, consequently, to reduce mortality in various pathologies [3]. MS is a powerful analytical technique that is used to identify unknown compounds and to quantify known compounds [4]. Mass spectrometry provides information concerning the molecular structure, atomic mass of whole molecules, molecular fragments, and atoms [5]. It has become a well-established tool for molecular biology, enabling the analysis of macromolecules and their interactions and modifications, organelles/cell lysates, intact cells/cell lines, tissues, and even organisms to characterize the protein structure under physiological or pathological conditions [6]. Consequently, biomedical MS becomes almost synonymous with proteomics and protein biomarkers [7], opening up large opportunities for “point-of-care” (POC) performances in the clinical settings [2]. Along with the well-known classical applications in biomarkers discovery, verification, and validation [8], MS-based diagnostic methods are involved today in the rapid and accurate identification of microbes [9,10], newborn screening [11], and quantification of therapeutic drugs [2]. MS is a powerful technique that detects the protein modifications in advanced personalized medicine, and the latest improvements in MS sensitivity and resolution allow the identification of new classes of tumor-specific proteoforms, including PTMs and variants originating from genomic and epigenomic aberrations [12].

MS-based methods analyze heterogeneous proteins, with the distinct identification of protein isoforms and their PTMs, by using only microgram (µg) quantities of biological materials [13]. A simple MS analysis is useful for the determination of proteins and peptide molecular weights by the detection of their molecular weight-related ions. A tandem mass spectrometer consists of two (MS/MS or MS^2^) mass analyzers connected by a collision cell [4] that takes peptides ionized in the mass spectrometer as intact molecules and breaks them into their constituents [7]. Consequently, the MS/MS technique is necessary for the accurate sequence analysis of peptide biomarkers [7] by the detection of precursor ions and product ions [14]. Liquid chromatography coupled with tandem mass spectrometry (LC-MS/MS) is a hyphenated technique for identifying small molecules, such as peptides [15], with high-throughput, speed, and resolution [16], combining the separation capability of high-performance liquid chromatography (HPLC) and the mass spectrometric ability of MS [14].

Here, we discuss the principles of tandem mass spectrometry, including experimental design; sample preparation; and sample analysis (fractionation, ionization, and analysis) and then discuss some applications of tandem mass spectrometry in biomedical research. 

## 2. MS/MS Proteomics Experimental Design

**Figure 1:**
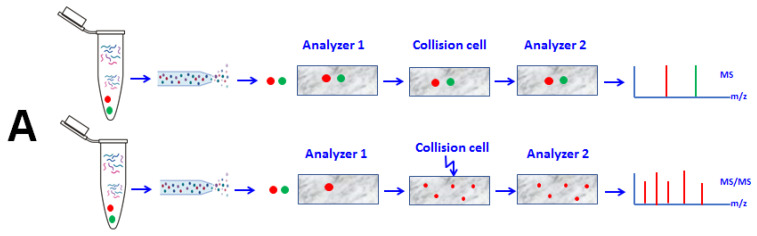

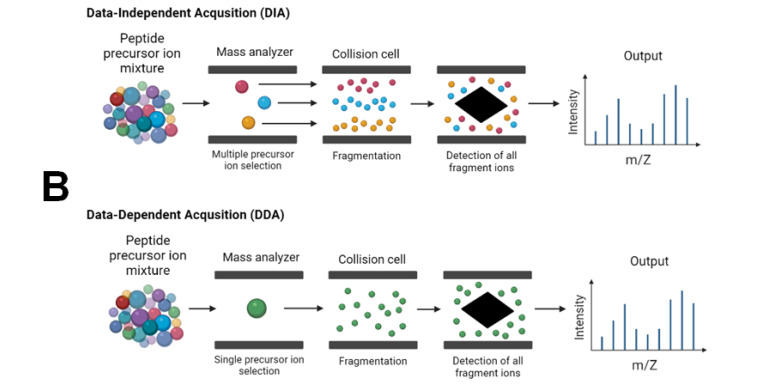
(**A**) Overview of analysis by Tandem Mass Spectrometry. The digested peptides are ionized and passed through the first mass analyzer; analyzed in the second analyzer; and detected as MS spectra, survey spectra, or MS1. The high-intensity peaks from the resulting spectra (MS1) are further selected in the first mass analyzer, fragmented in the collision cell, and then analyzed in the second mass analyzer, resulting in the product ions, detected in MS/MS spectra (MS2), which contain the information for peptide sequencing followed by protein identification. (**B**) Overview of the different modes of data collection in tandem mass spectrometry. In DIA, all precursor ions are analyzed simultaneously and in MS mode (i.e., with low-collision energy) and then fragmented in the collision cell using high-collision energy. Multiple product ions that resulted from the fragmentation of multiple precursor ions are then detected in one spectrum. In DDA, all precursor ions are detected in the survey MS spectrum, and then, the precursor ions that have the highest intensity are selected in the first analyzer (i.e., quadrupole), fragmented in the collision cell, analyzed in the second analyzer (i.e., TOF), and detected as the MS/MS spectrum.

Like any other MS proteomics experiment, the tandem mass spectrometry (MS/MS) proteomic workflow requires a succession of steps, starting with sample handling (collection, processing, storage, and tracking) and continuing with sample preparation, which includes sample pretreatment, extraction/separation of proteins, their digestion, purification, and fractionation of the resulting peptides, followed by data acquisition. The experimental design ends with data analysis, including database search, spectral library search, and de novo peptide sequencing [17], finally leading to protein identification (Figure 1).

### 2.1. Sample Preparation

Usually, sample handling, collection, processing, storage, and tracking involve the implementation of standard operating procedures [8]. Sample preparation is a critical step for proteomic studies [18], limiting the translation of proteomics techniques from research to routine clinical applications [19]. Well-known as sources for the discovery of new candidate protein biomarkers in biomedical research, body fluids, such as whole blood/plasma/serum [20], urine [21], milk [22], saliva [23], human eccrine sweat [24], bile [25], gingival crevicular fluid (GCF) [26], synovial fluid [27], tear biofilm [28], amniotic fluid (AF) [29], cervicovaginal fluid (CVF) [30], feces [31], exhaled breath condensate (EBC) [32], follicular fluid (FF) [33], aqueous humor [34], and cerebrospinal fluid (CSF) [35] or even primary cell culture conditioned media [36,37], as well as cell lysates [38] containing as many as several thousand proteins, can be analyzed with tandem mass spectrometry [39]. Dried nipple aspirate fluids on Guthrie cards/spots [40] and dried blood spot (DBS) proteins [41] are also analyzed by LC-MS/MS in clinical proteomics. LC-MS/MS proteomics had recent applications in forensic science focused on human samples, including hair [42], bone, body fluids, fingernail, muscle, brain, and fingermarks [17]. Compared with biological fluids that can be directly analyzed with LC-MS/MS, solid tissues, such as brain samples or others, must first be processed into a liquid form that can be analyzed by LC-MS/MS. This process involves additional steps, such as homogenization, microanalysis, ultrafiltration, and solid-phase microextraction (SPME) [43]. Proteomics based on tandem mass spectrometry also allows the taxonomical identification of microorganisms for investigating microbial diversity in various biological systems [44], as well as the identification of proteins at the single-cell level [45]. 

For samples consisting of cultured cells, washing with phosphate-buffered saline (PBS) or a similar solution is necessary before cell harvesting in order to remove contaminants and other proteins found in the culture medium [46]. In order to break the cells and extract the proteins, a lysis step is applied, usually performed by chemical lysis, using a lysis buffer containing strong denaturants (such as urea), ionic detergents (such as sodium dodecyl sulfate/SDS or sodium deoxycholate/SDC), or nonionic detergents (such as Triton X-100) compatible with MS, as well as mechanical lysis/sonication using a sonicator. In many cases, samples should be solubilized in a buffer solution as well. After lysis and solubilization, proteins are extracted or purified by the removal of contaminants. Further sample preparation may include dilution, protein precipitation (PPT) by organic solvents, such as ethanol/acetone/acetonitrile (ACN), or by their mixture with acids (e.g., trichloroacetic acid/TCA) or methanol/chloroform, which has been demonstrated as the best method for urine proteins extraction for LC-MS/MS [47], liquid–liquid extraction (LLE), phospholipid removal, supported or solid-supported liquid extraction (SLE), solid-phase extraction (SPE), absorptive chemistry (ACP)-based extraction [48], and automated on-line solid-phase extraction (SPE) [49]. To increase the accuracy of a proteomic analysis, body fluids such as human plasma, serum, or CSF can be depleted of highly abundant proteins, such as albumin and antibody components [50] that can be done based on dyes or by immunodepletion with antibodies [16]. Sensitive microdosing and DBS LC-MS/MS analysis require a specific online sample dilution, clean-up, and enrichment technique [51]. Consequently, many methods are also used to assess the concentration of proteins in the sample.

Complex protein mixtures may be separated using different technical approaches [52]: polyacrylamide gel electrophoresis (PAGE) in a single dimension based on the molecular weight of proteins (1-DE or SDS-PAGE) or by two-dimensional electrophoresis (2-DE), where the first dimension separates proteins based on their isoelectric point and the second dimension separates them by their molecular mass. Proteins can also be separated or fractionated based on their size/mass, charge, or hydrophobicity.

**Figure 2:**
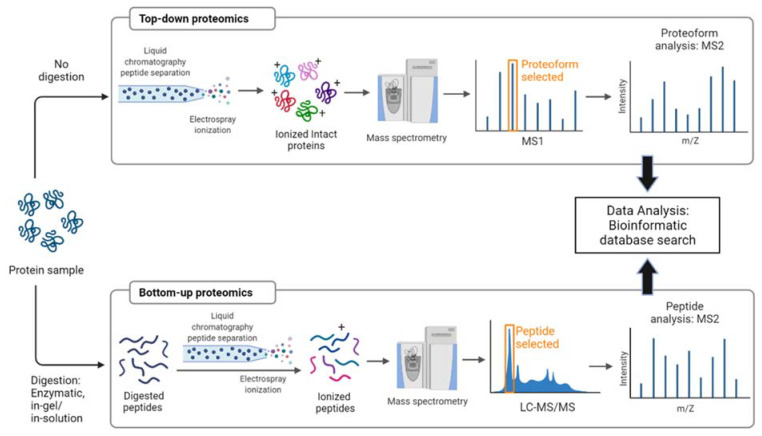
Schematics of top-down and bottom-up proteomics analysis.

There are two main approaches for MS-based proteomics: top-down [53] and bottom-up analyses [54], illustrated in Figure 2. The top-down proteomics analyzes intact/whole proteins without prior digestion of proteins into peptides [55] and assures a high coverage of genetic variants [56]. Additionally, this approach allows the analysis of PTMs at the intact protein level [57]. A relatively new approach is middle-down proteomics, where the proteins are subjected to limited proteolysis, followed by MS analysis. This is particularly useful in identifying coexisting PTMs. Bottom-up, middle-down, and top-down proteomics can also be classified based on the size of (or number of amino acids in) the peptide/mini-protein/protein. In bottom-up proteomics, the peptides are usually up to 3 to 4 kDa in size; in middle-down proteomics, peptides from 3 to 4 kDa up to 10–12 kDa are used, and in top-down proteomics, intact proteins and protein complexes are used. Applications of these types of proteomics are, however, different. While bottom-up proteomics can be used for the identification of a peptide, protein, and PTM in a peptide/protein, as well as quantitative proteomics, it provides very little information in the structure (and sometimes function) of that protein. Compared to bottom-up proteomics, top-down proteomics can provide a wealth of information on the structure of that protein. However, when a protein of a known mass is investigated, and it is modified by an unexpected PTM (i.e., truncation), data analysis may be difficult, because there is no match between the theoretical calculated mass of that protein and its experimentally determined mass. Furthermore, if the protein analyzed is unknown (and contains some PTMs like glycosylation and disulfide bridges) and the fragmentation in MSMS is not sufficient, then the data analysis may be challenging. Middle-down proteomics, as the name suggests, places this method between top-down and bottom-up proteomics. The more common bottom-up proteomics approach requires many preparation steps for protein digestion. Firstly, denaturing proteins disrupts the tertiary structure of proteins for the easy access of proteases. The next step is the enzymatic proteolysis by a protease such as trypsin (cleaves at the C-terminus of arginine and lysine), the most used enzyme in proteomics, chymotrypsin that targets primarily aromatic residues; lysine-sensitive (LysC), AspN, and GluC that target acidic amino acid residues; and proteinase K that is used to detach surface-exposed loops from proteins in membrane vesicles [58]. Chemical digestion by CNBr/formic acid also results in a collection of digested peptides, prior to MS analysis [52]. Widely used are in-gel [59], in-solution [60], and on-bead digestion [61] methods for protein analysis by MS. In-gel digestion (IGD) of gel-separated proteins for internal sequence analysis follows after 1-DE or 2-DE [62]. SDS-PAGE followed by IGD of the whole gel or the gel-excised slices is a popular method in MS, known as GeLC-MS/MS-based proteomics [63]. For more control over the outcome of the process, in-solution digestion (ISD) is preferred [60]. For the identification of more proteins of low abundance in a barley leaf sample by LC-MS/MS-based sequencing and the quantification of peptides, on-spin filter-aided digestion-based protocols provided a higher efficiency than the ISD protocols [18]. On-bead protocols can be also used prior to LC-MS/MS analysis; in these approaches, protein denaturation and trypsin digestion are performed directly on the affinity-bound complex on the magnetic beads, avoiding detergents and reducing sample loss [61]. Usually, the resulting peptides have an average size of 800–2000 Da, which is the preferred mass range for MS/MS sequencing [17].

Preparation of peptide samples for MS analysis also includes phosphopeptide enrichment methods and peptide online or offline sample clean-up/purification prior to MS analysis, removing excess salts and detergents, which interferes with further peptide ionization, and adding a chemical noise or background in the mass spectra [52]. After peptide purification, the samples containing peptides are lyophilized or vacuum-dried, followed by an optional fractionation for decreasing the peptide mixture complexity prior to LC-MS/MS analysis [17].

### 2.2. Sample Analysis (Fractionation, Ionization, and Analysis) and Data Acquisition

#### 2.2.1. HPLC/UPLC-Based Fractionation Prior MS Analysis

After sample preparation is finished, the peptides mixtures can be analyzed by an LC-MS/MS system, which is usually a conventional high-performance liquid chromatography (HPLC) or a modern ultra-high performance liquid chromatography (UHPLC) system, to separate the extracted peptides from the prepared biological samples [43]. The separation may be based on a variety of properties such as net charge (Ion-Exchange Chromatography (IEX)) or hydrophobicity (Reversed-Phase Chromatography (RP-HPLC)) [54]. To avoid the formation of sodium adducts of peptides, IEX is used only in 2D-HPLC, and it is always followed by RP-HPLC. The LC system is directly coupled to online ESI or indirectly using off-line MALDI with a tandem mass spectrometer for the detection and quantitation of various analytes. 

LC-MS/MS is an important technique for the identification and quantification of small molecules, especially from body fluids [49]. LC-MS/MS-based proteomics is a powerful tool based on the amino acid sequencing of peptides by inducing their fragmentation for identifying and quantifying proteins in biological samples [64]. This technique, as well as 2-DE, are widely used in clinical medicine [65], both being successfully used for biomarker discovery [7]. Peptides separated by HPLC are then fragmented by different fragmentation methods, such as collision-induced dissociation (CID)/collision-activated dissociation (CAD), electron ionization dissociation (EID), electron capture dissociation, and electron-transfer dissociation (ETD) [66], and the MS/MS spectrum for each fragmented peptide is recorded. Consequently, there are up to several thousand MS/MS spectra for each complex biological sample. Each MS/MS spectrum is used to search protein databases for matched peptides, a protein being identified often by multiple sequenced peptides from the same protein.

Technically, HPLC-based separation and then the ESI-MS-based analysis of peptides mixtures became the norm in the largest scale proteomics experiments. While the mass spectrometer can have a particular speed in acquiring data, in terms of time spent for a MS survey scan and number of MS/MS per scan (in DDA mode) or the time spent in high energy (DIA or MSE mode), the more important component is the HPLC fractionation. For example, when a peptides mixture that resulted from digestion of a serum sample is run in a 20- or 60-min HPLC gradient at a high flow rate (i.e., 200 µL/min), only a few proteins will be identified. However, if the gradient time is extended to 120, 240, or 360 min, then more proteins will be identified. Furthermore, if the flow rate is decreased to nanoflow mode, then the HPLC will deliver the peptides at a slower rate, thus allowing the MS to analyze more ions and then identify more peptides. Therefore, while many people focus on the high-end MS, the more important factor is the HPLC separation of these peptides—in particular the gradient slope and length—and the HPLC’s flow rate.

Usually, the proteome-wide studies are based on 2D-LC-MS/MS, but thousands of proteins can be identified in a single-run nano-LC-MS/MS experiment using ultra-long gradients for protein analysis from whole-cell extracts, such as HeLa cell lysates [67]. Data acquisition in a single-shot approach by µLC-MS/MS has become a current trend in proteomics that sometimes it allows the identification of 7000–9000 proteins from cell lines and tissues [68]. Additionally, to increase the number of identified peptides, one tissue section can be analyzed by MALDI-MSI that can produce thousands of ion images, while an adjacent tissue section is homogenized and analyzed by LC-MS/MS [69]. It must, however, be emphasized that the number of proteins identified in a proteomics experiment does not reflect in any way the sequence coverage of these protein. A protein can confidently be identified by minimum two peptides, which can provide a coverage of that protein from 10% for small proteins, to less than 1% for large proteins. Yet the full structural characterization of a protein (i.e., post-translational modifications) requires full coverage of that protein. Therefore, the number of proteins in a large-scale experiment should be viewed only from the investigator’s perspective: as a number of proteins, with only partial coverage, and no true PTM analysis.

#### 2.2.2. Sample Ionization Using MALDI-MS or ESI-MS

After sample preparation, the peptide mixture is ionized by a soft ionization method, such as MALDI-MS or ESI-MS. The analysis of a wide range of large biomolecules including proteins is based mainly on MALDI-time-of-flight (TOF)-MS and ESI-MS [7]. Classic ionization methods, such as electron ionization (EI) and chemical ionization (CI), are not used often in clinical diagnostic assays, because they are considered harsh ionization methods [2]. Development of new types of soft ionization techniques, such as ESI and MALDI, has increased the size of protein molecules that can be studied, as well as the diversity of MS applications in biology and medicine [13], both being the core of a revolution in MS [7]. MALDI-TOF MS has a wide spectrum of applications in research and clinical laboratories for biomarkers discovery, validation and monitoring in oncoproteomics, neuroproteomics, parasitoproteomics, metaproteomics, age-related diseases, non-tumoral pathology assessment, identification and characterization of bioactive molecules, structural biology, developmental biology, genetic disorders, identification of clinical pathogenic anaerobic bacteria [9], using either microbial intact cells or cell extracts [70], behavioral studies, forensic medicine, food industry, paint and cultural heritage analysis [71], toxicology and ecotoxicology, pharmaceutical research, etc. [38]. 

In MALDI-MS, the sample for analysis is digested with a proteolytic enzyme, usually trypsin. The resulting mixture of peptides is spotted with an energy-absorbent organic matrix onto the metal MALDI target, such as alpha-cyano-4-hydroxycinnamic acid (HCCA) or 2,5-dihydroxybenzoic acid(DHB), air-dried for matrix crystallization and sample co-crystallization. Further, the target is irradiated with a laser beam, followed by the desorption ionization of the analytes, resulting in singly protonated ions from the analytes in the sample [70]. The protonated/deprotonated ions (positive or negative ionization mode) are separated by their *m/z* and the charged analytes are detected and measured using different types of mass analyzers, such as time-of-flight (TOF), quadrupole mass analyzers, ion trap analyzers, etc. A characteristic ion spectrum is generated for analytes in the sample. An application of collision-induced fragmentation of amino acids and their derivatives by MALDI TOF/TOF tandem MS with applications in clinical medicine was first published in 2007 [72].

ESI-MS is an important ionization technique applied in clinical laboratories [5]. It is performed in solution; the sample is sprayed into the MS and as the droplets evaporate, electrical charges are transferred to molecules present in the droplet and multiple charged ions are produced, and the mass analyzers generate complex ESI spectra [7]. Coupled with an HPLC system prior to MS analysis and with the additional separation capabilities of tandem MS, the HPLC/ESI-MS/MS has become a rapid analysis technique with high sample throughput [5]. LC-ESI-MS/MS analysis of proteins from blood plasma has identified and quantified new diagnostic and therapeutic peptides in multiple clinic populations [73].

Thus, the resulting peptides separated by LC/HPLC are analyzed by MS/MS or MALDI-TOF-MS, as well [55], and serve as unique identifiers of the fingerprint of the protein [74]. Bottom-up proteomic analysis proves high specificity, accuracy, and throughput, but may result in an incomplete protein coverage, biases in quantification, and difficulties in the proteoform analysis [75].

#### 2.2.3. Data Acquisition

Data acquisition may be performed by non-targeted MS/MS and targeted MS/MS. In non-targeted/shotgun MS/MS, the ions (that correspond to peptides) with the highest intensities are isolated and fragmented and MS/MS spectra are recorded [17]. This is usually done using Data-Dependent Acquisition (DDA), but Data-Independent Acquisition mode (DIA) can also be used [76]. The DDA method selects and puts forwards for subsequent fragmentation certain precursor ions that correspond to peptides generated during the first cycle of MS (M1), also called MS survey. Usually, these are the precursors of highest abundance, and the low abundance precursor ions may not be selected for fragmentation (and do not have a corresponding MS/MS). In the DIA approach, all peptides/all detected precursor ions generated during the first MS cycle can be fragmented using high collision energy and the fragment ions are accumulated in a fixed number of wide isolation windows that span the entire *m/z* range [77]. DIA-MS is a next-generation proteomic methodology with highly reproducible analysis of cellular and tissue specimens and better sensitivity [77].

When a mass spectrometer capable of MS/MS in DDA mode, using soft ionization techniques (ESI or MALDI), is coupled with a LC/HPLC/UPLC, this will allow untargeted detection of a large number of unknown analytes with high sensitivity [78]. Specifically, all MS and MS/MS spectra that are detected in a DDA experiment can be used for database search and peptide (and protein) identification (and sometimes label-free protein quantitation) and then the MS parameters of the precursor and product ions of these peptides (elution time, charge state, amino acid sequence, intensity of the fragment ions in MS/MS spectra) can then be used for developing targeted proteomics methods (i.e., SRM or MRM methods; see below). 

**Figure 3:**
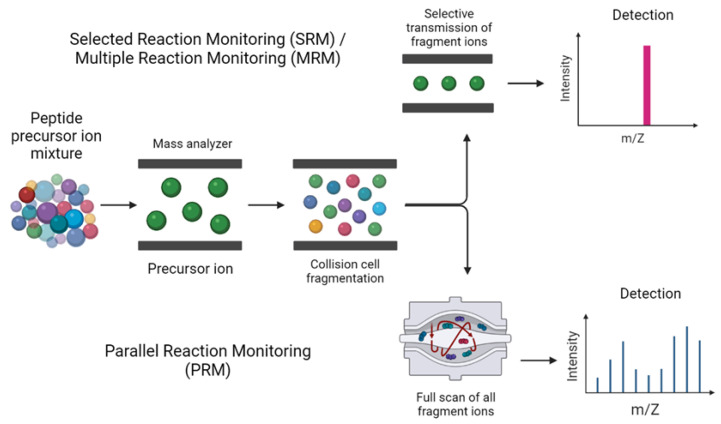
SRM/MRM analysis showing single ion transitions being monitored in comparison to PRM, where a single ion is fragmented from several fragment ions.

Targeted LC-MS/MS-based proteomics detects and quantifies a small number of peptides that are specific for a preselected group of proteins of interest, when prior information about those peptides is available from the DDA methods (elution time, charge state, the highest intensity product ions, etc.). Good examples include Selected or Multiple Reaction Monitoring MS (SRM-MS/MRM-MS) [79]. In quantitative proteomics and biomarker discovery [80], SRM-MS/MRM-MS (Figure 3) is one of the most used MS-based targeted methods [81] that utilizes a triple quadrupole (QqQ) instrument or a hybrid quadrupole-linear ion trap (QTrap) mass spectrometer [82]. In QqQ, an ion of a particular mass is selected in the first mass analyzer (MS1/Q_1_), fragmented into a collision cell (Q_2_),resulting in specific product ions, and then analyzed in a second mass analyzer (MS^2^/Q_3_) for detection [83]. Thus, the first and the third quadrupoles act as mass filters for the precursor (Q1) and product (Q3) ions. While most SRM or MRM experiments are performed on triple quadrupole instruments, recent advances in QTOF technology also allows for QTOF to do DDA or DIA and then to MRMs (i.e., Xevo G2-XS instruments). The targeted experiments known as Parallel Reaction Monitoring (PRM; Figure 3), where a list of precursor peptide ions are selected for fragmentation followed by the detection of a few or most major product ions, can be performed on hybrid quadrupole-Orbitrap and quadrupole time-of-flight (QTOF) mass spectrometers, with PRM providing a high specificity because a full MS/MS spectrum is acquired in high-resolution mode, containing all potential product ions and confirming the identity of the target peptide [82].

### 2.3. Data Analysis

Peptide fragments are analyzed for *de novo* peptide sequencing, peptide mass fingerprinting (PMF) [74], or amino acid sequencing of the peptide, followed by protein database search of the resulting fragments [84]. The identification of the unknown protein is done by matching the resulting multiple peptide masses, that allow more extensive coverage of the protein, with the theoretical peptide masses generated by the in silico digestion on database proteins with the same enzyme used in the experimental digestion [74]. Many search engines are available, i.e., Mascot, X!Tandem, ProteinLynx Global Server, Proteome Discoverer [38], MS-Fit, ProFound [74], etc., which use different strategies for database search.

After database search and data analysis, proteins are identified, characterized and quantified, followed by data verification, validation, and interpretation [55]. Usually, the differential expression of proteins identified during the discovery step is verified/validated by performing an enzyme-linked immunosorbent assay (ELISA). Western blotting can be used to confirm protein identification [74]. 

Usually, identification of a peptide based on only one high quality MS/MS spectrum is sufficient for identification of that particular peptide and then the protein that peptide is part of. Identification of two or more peptides that are part of the same protein usually allows identification of that protein with higher confidence. Therefore, while one peptide could suffice to identify a protein, to avoid false positive identification, most researchers try to use two peptides per identified protein, which is also required by most proteomics journals. To increase the protein coverage, several database search strategies can be used, each of them with their advantages and disadvantages. Increasing the window error for the precursor ion and fragment ions during database search allows identification of more peptides and proteins. Similarly, identification of more peptides by using additional post-translational modifications as variable modifications, increasing the number of trypsin missed cleavages, or using one 13C can also lead to identification of more peptides, but each additional search leads to extra time needed for the analysis. Furthermore, post-acquisition analysis of the data using additional software such as Scaffold allows flexibility in additional data interrogation without consuming the investigator’s time.

A more specialized approach in data analysis of tandem MS data is when a protein characterization is needed. This is the case when one analyses a protein for structural biology studies in basic research or for full characterization and stability studies of proteins for applied research. In these situations, full protein coverage is needed, from both ESI-MS and ESI-MS/MS analysis, as well as LC/HPLC//UPLC-MS/MS analysis.

## 3. Applications of Tandem Mass Spectrometry in Biomedical Research

### 3.1. Applications of Tandem MS-Based Proteomics in Systemsbiology

MS-based methods are used for proteome-wide structural studies, MS emerging as a significant tool for systems biology that interrogates the complex biological systems, from subindividual to ecological levels of organization, through large-scale identification and quantification of various biomolecules [85], including proteins and peptides.

Considering ecological/supraindividual levels of organization, such as population and community, proteomics investigation based on tandem MScan lead to a deeper understanding of molecular processes occurring in a biological system, such as microbial communities or microbiota that reside in gut [86], on skin [87], and vagina [88]. LC-MS/MS also identified a series of proteins in blood plasma of freshwater and seawater life stages of rainbow trout (*Oncorhynchus mykiss*) [89] to emphasize the mechanisms of adaptation of organisms to their environmental variable conditions.

Organism-level systems biology aims to identify, analyze and characterize molecular and cellular networks with various biological functions in whole organisms [90]. LC/HPLC/UPLC-MS/MS-based proteome profiling was established to characterize, classify, and identify microorganisms [91], as well as to design the whole embryo proteomes to decipher the developmental processes of usually used animal models, such as *Caenorhabditis elegans* [92], sea urchins [93], fruit fly *(Drosophila melanogaster*) [94], zebra fish (*Danio rerio*) [95], or African clawed frog (*Xenopus laevis)* [96].

LC-MS/MS-based proteomics identified the differentially expressed proteins at organ level, such as mouse stomach [97], human heart [98], mouse [99] and human livers [100], skeletal and cardiac muscles [101], kidneys [102], mouse [103] and human [104] lens, rat brains [105] and human brains in Alzheimer’s disease [106], and rat sciatic nerve during the regeneration process [107]. LC/UPLC-MS/MS was also used to study the skin annexes proteomes, such as hair and eccrine sweat glands, detecting the main proteome variation at different body locations of hairs, based on genetically variant peptide detection for protein-based human identification [42], as well as the interindividual variability of sweat secretion parameters as a promising biofluid analysis for non-invasive monitoring and personalized medicine [24]. Human skin barrier-related proteins were non-invasively sampled by 3M medical adhesive tapes and further identified and quantified using the LC-MS/MS analysis, with applications in the study of skin aging and various skin diseases related to epidermal barrier destruction [108]. Consequently, LC-MS/MS can identify protein alterations associated with age and age-related diseases [109]. The proteomic profiles obtained by tandem mass spectrometry are usually correlated with the specific developmental stages, as well as with spatial and temporal structural heterogeneity of organs investigated under normal function or pathological condition.

The proteomic analysis of tissue systems is important for understanding of molecular mechanisms that differentiate between healthy and disease states [110], as well as for identification of specific proteins in various tissues in correlation with their biological functions. The proteomics-based investigation of epithelial tissues using LC-MS/MS allowed the identification and characterization of proteins in human olfactory epithelial tissue [111], intestinal epithelial cells [112], epidermis [108], endothelial cells [113], endometrial luminal epithelial cells responsible for embryo implantation [114], human corneal cells infected with HSV-1 in herpes simplex keratitis [115], or human mammary epithelia [116]. Skeletal muscle tissue was analyzed using LC-MS/MS emphasizing the impact trauma in rats [117]. Cardiac muscle proteomic analysis based on tandem MS revealed cardiac adaptation mechanisms to regular exercise [118].

Cell-level systems biology uses LC-MS/MS proteomics-based strategies to determine the abundance, modification state, and localization of proteins from different cell organelles, such as the plasma membrane (PM), cytosol, nuclei, mitochondria, endoplasmic reticulum (ER), lysosomes, peroxisomes, cytoskeleton, and exosomes [119]. The MS-based shotgun proteomics was used to identify the complete proteomes from a single-cell type, such as HeLa cells [120], single lens fiber cells [121], and human T cells [122], as well as single embryonic cells [123]. Molecular dissection has become an integral part of structural studies in biology. Dissection of ribosomes throughout the kingdoms of life has been possible by a three-level hybrid MS approach that included shotgun LC-MS/MS, which enabled the identification and quantification of ribosomal proteins and their PTMs in top-down LC-MS/MS, which provided information on proteoforms of ribosomal proteins that have been lost upon tryptic digestion into peptides, and native MS, which introduced the intact ribonucleoprotein complexes into the mass spectrometer, assuring their accurate mass measurements and identifying their composition [124]. LC-MS/MS and MALDI-TOF-MS were used to profile human sperm cell proteins to identify defective molecular pathways in reproductomics and to discover new candidate biomarkers in the diagnosis and therapeutic strategies of male infertility [125]. LC-MS/MS identified novel protein targets in the secretome that is involved in cell-to-cell communication [55]. Additionally, LC-MS/MS is an efficient technique to identify proteins with specific functions from individual human oocytes to investigate their biology and preimplantation development [45]. Proteins from human dendritic cells were analyzed by an optimized protocol using a triple TOF mass spectrometer [126]. A shotgun analysis by LC-MS/MS-based proteomics of secreted proteins of human adipose-derived mesenchymal stem cells has been performed for clinical applications of liver cell therapy using stem cells as an alternative to liver transplantation [14].

### 3.2. Applications of Tandem MS in Biofluids Proteomics and Enzyme Activity Assessment

Biofluid analysis represented a challenge to proteomics as they are often present only in small amounts and can express very different concentration of proteins [127]. However, proteomic approaches are essential for the discovery of biofluid biomarkers. Consequently, tandem MS-based protocols were widely used for biomarkers discovery in various biofluids. For example, proteins from human whole saliva were identified using LC-MS/MS [23]. Human and non-human proteins were identified in human milk samples, where all peptides were separated and analyzed using an HPLC system coupled online to a hybrid quadrupole-Orbitrap mass spectrometer [22]. The proteins identification in urine of healthy human was performed using a triple TOF mass spectrometer coupled with a UPLC system [21]. The depth characterization of venom proteome of honeybee has been realized using LC-MALDI-TOF/TOF tandem mass spectrometry and LC-ESI-QTOF MS/MS in order to emphasize the role of toxins, allergens, and other protein components in the envenoming process, including discovering substances with potential pharmacological activity [128]. Protein identification from dried nipple aspirate fluid on Guthrie card collections is possible using nLC-ESI-QTOF analysis, with potential applications for early breast cancer detection and classification [40]. The human cerebrospinal fluid (CSF) proteome was described using different strategies, including the Orbitrap LC-MS/MS analysis [35]. A nanoLC-MS/MS approach identified proteins in bile, which opened new windows to identify new biomarkers for cholangiocarcinoma, hepatocellular carcinoma, and pancreatic cancer for the improvement of patients’ clinical surveillance [25]. The overexpressed and downregulated proteins in cervicovaginal fluid (CVF) have been identified in preterm birth (PTB) compared to full-term birth, suggesting a potential to discover candidate biomarkers for asymptomatic PTB, using iTRAQ coupled with 2D-nLC-MS/MS [30]. By Tandem mass tag (TMTs) quantitative mass spectrometry analysis of exhaled breath condensate identified the differentially expressed proteins in patients with stable chronic obstructive pulmonary disease (COPD) compared with controls [32]. Many differentially expressed proteins were identified in human follicular fluid (hFF) from over-weight/obese and normal weight patients with polycystic ovary syndrome (PCOS), using tandem mass spectrometry [33]. 

Tandem mass spectrometry combined with liquid chromatography was applied for multiplex newborn screening of lysosomal enzymes in dried blood spots (DBS) for mucopolysaccharidosis and type 2 neuronal ceroid lipofuscinosis [129]. LCMS/MS can be also used for quantification of activity of human intestinal cytochrome P450 (CYP) and uridine 5’-diphosphate-glucuronosyltransferase (UGT) involved in first-pass metabolism of many drugs with oral administration [130]. Proteolytic enzymes of the carnivorous pitcher plant (*Nepenthes* spp.), such as nepenthesin, neprosin, and prolyl endoprotease, were identified by LC-MS/MS in order to be used to alleviate the human deficiency in proteolytic enzymes in celiac disease that is triggered by partially digested gluten proteins [131]. Host plant enzymes, which influence the midgut protein content of phytophagous insects, with an important role in plant protection against herbivores [132], were identified using LC-MS/MS. For efficient biological control of pests and management of insect resistance, LC-MS/MS is useful to identify novel candidate insecticidal proteins [133].

### 3.3. Applications of Tandem MS in Biomarkers Discovery

LC-MS/MS-based methods are applied in various important diagnostic protocols of laboratory medicine [134], with wide utility especially for identification and quantification of non-invasive biomarkers, using bodily fluids for diagnosing diseases. Plasma or serum may be used to detect inflammation, oxidative stress (OS), and tumoral biomarkers of colorectal cancer [135] for the early identification of diagnosis and metastasis biomarkers in non-small-cell lung cancer patients [136], as well as for the identification of proteins secreted into the proximal fluid and membrane proteins. Proximal fluid and membrane proteins are often glycosylated and released into the plasma/serum of breast cancer (BC) patients [137] or secreted by different BC cell lines [138]. Plasma proteomic changes identified by TMT isobaric labeling combined with nanoLC-MS/MS with an ESI ionization nano-spray source represent potential biomarkers for early detection of women at risk for gestational diabetes mellitus (GDM) [50]. LC-MS/MS was used to discover a putative plasma protein biomarkers panel for acute-on-chronic liver failure (ACLF) induced by hepatitis B virus (HBV) [139], as well as to discriminate between the clinicopathological profiles and outcomes of patients with chronic HBV genotype B infection and those with chronic HBV genotype C infection by DIA-based quantitative LC-MS/MS serum proteomics [140]. One-dimensional LC-MS/MS can quantify changes in the nipple aspirate fluid (NAF) proteome associated with BC development [141]. The comprehensive proteomic analyses of saliva samples by using LC-MS/MS led to discovery of potential non-invasive biomarkers for oral cancer (OC) [142], canine oral squamous cell carcinoma [143], and gastric cancer (GC) [144]. NanoLC-MS/MS applied to whole saliva and gingival crevicular fluid (GCF) showed that both fluids contain important biomarkers with multiple applications in dentistry and medicine [26]. LC-MS/MS analysis of whole saliva is a powerful tool to characterize the changes associated with periodontitis [145]. Urine is also a useful noninvasive source of potential biomarkers in the detection of breast cancer, a list of novel overexpressed proteins being detected by LC-MS/MS analysis [146]. Label-free LC-MS/MS tear film proteome analysis has revealed new putative biomarkers and therapeutic targets in ocular surface diseases, such as dry eye and Meibomian gland dysfunction [28].

In rheumatic diseases, such as ankylosing spondylitis, the label-free LC-MS/MS study identified differentially expressed proteins in the synovial fluid that may serve as diagnostic or prognostic biomarkers [27]. A MALDI-TOF MS protocol was used to identify a panel of salivary biomarkers for temporomandibular joint disorders [147].

A label-free nanoflow UHPLC-MS/MS showed that fetuin-A could be a potential aqueous humor protein biomarker associated with diabetes mellitus (DM) and smoking as cataract risk factors [34]. LC-MS/MS proteomic analysis identified overexpressed proteins in blood samples of patients with type 2 DM, which could be involved in the pathogenesis of this disease or serve as disease biomarkers [148].

The MS approaches are also employed in the studies for pregnancy-related complications [149]. LC-MS/MS of amniotic fluid (AF) from women with preterm labor (PTL) identified new biomarkers and specific protein pathways related to spontaneous preterm delivery [150]. The placental protein 14 (PP14) may be a novel potential biomarker for premature rupture of membranes (PROM) that was identified by investigation of proteome profiles of AF and maternal plasma that was examined by LC-MS/MS proteomic techniques [151]. The transfer of multiple protein variants between mother and fetus across the placenta was investigated by in-depth human plasma proteome analysis based on high-resolution isoelectric focusing (HiRIEF) LC-MS/MS [152]. The protein profiles of clinical bone marrow mononuclear cells (BMMC) samples from patients with immune thrombocytopenia (ITP) was reported by an LC-MS/MS study that identified differentially expressed autophagy-related proteins, concluding that the disruption of autophagy pathway is a putative molecular and pathological mechanism of ITP [153]. In biomarker-guided precision pharmacotherapy, LC-MS/MS-based proteomics identified the protein abundances of many drug-metabolizing enzymes and transporters in tissues [64]. Most studies used a MS/MS proteomics approach to identify key blood biomarkers in preterm neonates [154], such as those associated with retinopathy of prematurity [155], oxidative stress in neonatal lung diseases [156], such as bronchopulmonary dysplasia (BPD), prediction and early diagnosis of necrotizing enterocolitis (NEC) [157] and late-onset sepsis [158], and gestational age [154]. An increased risk of cardiovascular diseases in adulthood may be assessed using GeLC-MS/MS-based urinary proteomics in preterm infants, which allows identification of candidate biomarkers [159]. 

### 3.4. Applications of Tandem MS in Oncoproteomics and Non-Oncologic Diseases

LC-MS/MS is a useful technique to identify and quantify proteins in cancer cell lines and human tumors [160]. In breast cancer patients, LC-MS/MS-based targeted proteomics provided useful information concerning the identification and quantification of proteins, including the interaction, function, network, and signaling pathways associated with tumorigenesis, clarifying the different stages-specific protein pathways [161], metastatic mechanisms [162], and hypoxia-related biomarkers that promote cancer progression [66]. Super-stable isotope labeling of amino acids in cell culture (SILAC) and LC-MS/MS were used to verify the reliability of the proteomic data obtained using sequential windowed acquisition of all theoretical fragment ion mass spectra (SWATH-MS) in nasopharyngeal carcinoma [163]. Differentially expressed proteins during TGF-β-induced EMT might be assessed by a complex analysis including temporal quantitative proteomics by iTRAQ 2D-LC-MS/MS that identified post-transcriptional modulation of actin-cytoskeleton regulators [164]. 2DE-LC-MS/MS identified many proteins in each 2D gel spot in analysis of complex human pituitary adenoma tissue [165]. The LC-MS/MS was used to identify proteins in serum and urine of patients in different stages of non-small-cell lung cancer (NSCLC) [136]. LC-MS/MS identified new protein biomarkers for cervical precancerous lesions and for prognostic evaluation of cervical cancer (CC) in plasma samples [166]. The isoform-1 of multimerin-1 (MMRN1) and leucine-rich α-2-glycoprotein (LRG1) were identified as highly expressed, while S100 calcium-binding protein A8 (S100A8), serpin B3 (SERPINB3) and a cluster of differentiation-44 antigen (CD44) were significantly downregulated in the urine of CC patients [167]. Another study used breast milk to identify differences in the proteomes of donors with breast cancer and matched controls [168]. Furthermore, a similar study also investigated the differences between milk proteomes of the breast with cancer and the other breast without cancer (as a matched control), thus eliminating the genomic differences (the same donor, just different proteomes due to cancer). Lactoferrins, caseins, fatty acid synthase, xanthine dehydrogenase and other proteins were among the dysregulated proteins that were also found by others dysregulated in the blood of donors with breast cancer and other cancers [168,169,170]. Nondestructive analysis for ex vivo and in vivo intraoperative cancer diagnosis using MasSpec Pen technology that involved tandem mass spectrometry analysis of human samples was used to characterize a variety of potential cancer biomarkers identified as metabolites, lipids, and proteins [171], allowing rapid diagnosis and tumor margin assessment that is critical for cancer surgery [172].

The advanced LC-MS/MS technique can explain the significant changes in intracellular signaling and protein or RNA metabolism in normal looking and lesioned psoriatic skin in order to explain the faster turnover and higher metabolic rate in psoriatic epidermal cells [173]. Proteins involved in the alteration of cytoskeleton and energy metabolism pathways have been detected by LC-MS/MS as differentially overexpressed or downregulated in the myocardial tissues of mice with coronary microembolization compared with the control group of sham-operated mice [174]. 

### 3.5. Applications of Tandem MS in Neuroproteomics

For quantitative analysis of the brain proteome, recent developments in DIA-tandem mass spectrometry are useful to decipher synaptic proteomes and neuronal plasticity mechanisms involved in basic brain research and clinical applications [175]. LC-ESI-MS/MS is useful to quantify neurotransmitters in brain tissue homogenates in experimental mice [176], as well as for identification, characterization, and quantification of specific neuropeptides in rat spinal cord in order to determine neuropeptide homeostasis and dynamics in animal neuropathic and chronic pain models [177]. Additionally, LC-MS/MS has applications in nonclinical studies involved in drug development, drug metabolism, and drug pharmacokinetics in the central nervous system (CNS) [178]. A highly sensitive LC-MS/MS protocol was introduced for the quantitative measurement of amyloid-β (Aβ) species in cerebrospinal fluid (CSF) for clinical evaluation of Alzheimer’s disease (AD) patients [179], as well as for quantifying plasma Aβ_1-40_ and Aβ_1-42_ peptides and apolipoprotein E (APOE) for the assessment of brain amyloidosis [180]. Phosphorylation and aggregation of neuronal-tau protein are known as hallmarks of cognitive deficits in AD and can be revealed by a combinatorial native MS and LC-MS/MS [181]. In Parkinson’s disease (PD) [182], amyotrophic lateral sclerosis (ALS) [183], as well as in neural tube defects (NTDs) [184], several new proteins, pathway alterations, and candidate biomarkers have been identified in CSF using label-free LC-MS/MS analysis [182]. In autism spectrum disorder (ASD), an LC-MS/MS-based proteomics study identified several differentially expressed proteins for discovering urinary [185] and salivary protein biomarkers [186]. The proteomic results obtained by HPLC-MS/MS and MALDI-TOF MS, validated by ESI-QTOF MS, were used to characterize potential plasma biomarkers for distinguishing patients with major depressive disorder (MDD) from patients with schizophrenia (SCZ) [187]. In diabetic painful neuropathy (DPN) studied in rats, a tandem mass tag (TMT) labeling approach coupled with LC-MS/MS was used to identify potential biomarkers in the spinal dorsal horn to understand the DPN pathogenesis and the mechanisms that explain the electro-acupuncture analgesic, antioxidant, and hypoglycemic effects [188]. 2DE combined with LC-MS/MS analysis of serum proteome may offer indications about the biological mechanisms of medication overuse headaches that share some biomarkers with chronic pain conditions [189]. Candidate protein biomarkers for burning mouth syndrome were also identified by a LC-MS/MS approach [190]. 

### 3.6. Tandem Mass Spectrometry Detection and Quantification of PTMs

Post-translational protein modifications (PTMs) play a key role in the regulation of all cellular processes in eukaryotic cells [191]. They occur after biosynthesis of proteins and change their structure, temporal-spatial localization, and molecular function by proteolytic processing of the polypeptide chain, addition or removal of chemical modifying groups to one or more amino acid residues [192], or by the introduction of covalent crosslink between domains of the protein [193]. Together, PTMs of human proteins and PTM-type-specific protein interaction networks (PINs) are involved in disease processes [194]. Tandem mass spectrometry is highly involved in the characterization of modified proteins by amino acid sequencing and specific detection of post-translationally modified amino acid residues [193]. Thus, methylated, phosphorylated, and acetylated proteins were detected by LC-MS/MS in patients with blood cancers [195], as well as the N-glycosylated membrane proteins identified as biomarkers in breast cancer cell lines [196]. LC-MS/MS also identified S-nitrosylated (SNO) proteins of endothelial cells in human umbilical vein [197], as well as ubiquitination sites [198], or ubiquitinated proteins, in various pathological conditions, such as human pituitary adenoma tissues [199], SKOV3 ovarian carcinoma cells [200], or *Saccharomyces cerevisiae* in response to oxidative stress [201]. The LC-MS/MS-based workflow is involved in quantification of multiple types of protein thiol PTMs that play an important role in redox-dependent regulation and signaling, enzyme activity, autophagy, unfolded protein response (UPR), protein ubiquitination pathway, and EIF2 signaling in pancreatic beta cells under ER stress [202]. Some PTMs, such as phosphorylation and acetylation, are strongly associated with neoplastic processes and have been performed using high-resolution UPLC-MS/MS in gender-specific ovarian or breast cancers [203]. LC-MS/MS is involved in the analysis of site and structure-specific core protein fucosylation that regulates many biological processes, such as protein folding, stability, immune response, and receptor activation, being associated with various diseases including autoimmunity and cancer, including liver cirrhosis [204]. 

**Figure 4:**
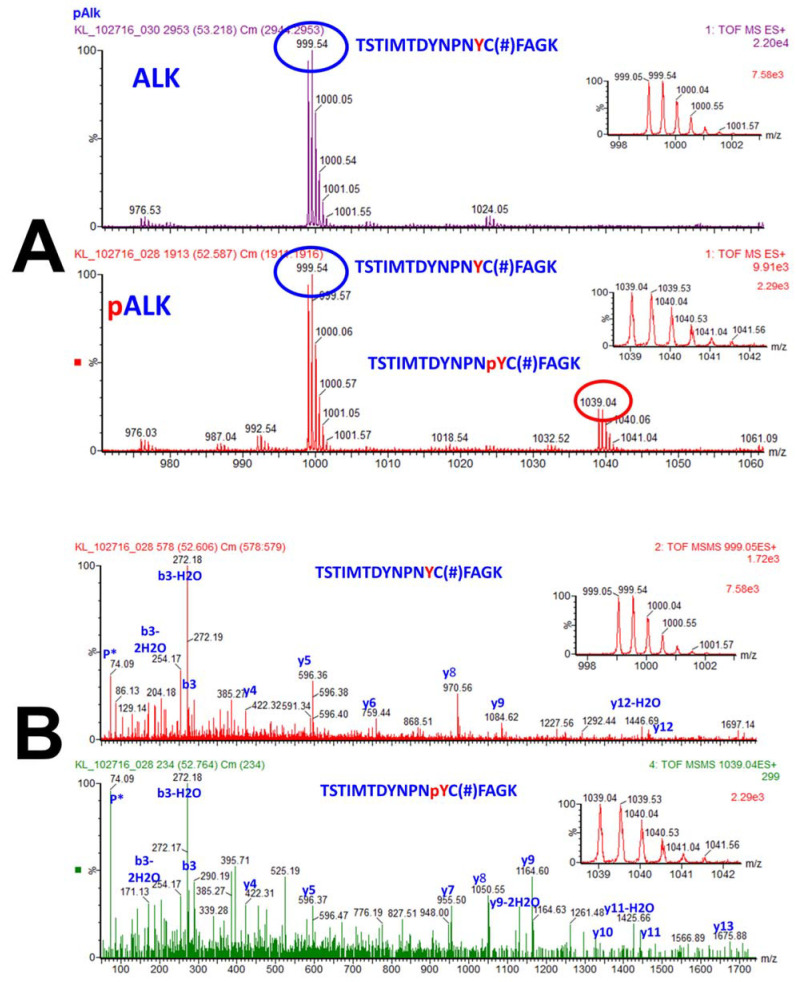
Identification and quantification of a phosphopeptide, and localization of a phosphorylation on a tyrosine residue within a peptide. (**A**): The MS spectra of the precursor ions with *m/z* of 999.05 (2+) and 1039.04 (2+) that correspond to unphosphorylated peptide TSTIMTDYNPNYC(#)FAGK and its phosphorylated counterpart TSTIMTDYNPNpYC(#)FAGK. Note that C(#) represents cysteine modified by acrylamide (propionamide) and pY corresponds to phosphorylated tyrosine residue. While the top spectrum contains only the unphosphorylated peptide, the bottom spectrum contains precursor ions that correspond to both unphosphorylated and phosphorylated peptides (and the ratio of the two precursor ions indicates the percentage of phosphorylation of that peptide). Note that the peptide discussed is part of ALK, anaplastic lymphoma kinase (ALK) protein. (**B**): The MS/MS spectra of the precursor ions that correspond to the unphosphorylated and phosphorylated peptide not only confirm phosphorylation of this peptide, but also the location of this modification. Note that this peptide contains two tyrosine, two threonine and a serine residue, all of which can be potentially phosphorylated, yet the phosphorylation happens at only one specific tyrosine residue. Fragmentation of the precursor ions in MS/MS produced a series of b and y fragment ions that correspond to the unphosphorylated (top) and phosphorylated (bottom) peptide. Simply identifying y8 and y9 as not phosphorylated (top MS/MS spectrum), and y7, y8, and y9 are sufficient to demonstrate that the tyrosine residue adjacent and upstream of cysteine residue is the phosphorylated amino acid. Furthermore, y10 and y11 demonstrate that the next tyrosine upstream is not phosphorylated, and the *m/z* of the y13, b3, and the precursor ion indicate that none of the three serine and one threonine residues are phosphorylated. Reproduced from Reference [205].

Figure 4 reflects a showcase of the power of tandem MS for both identification and quantitation of a peptide and a phosphopeptide (tyrosine phosphopeptide), as well as the exact location of the phosphorylation within the peptide sequence, even more than one tyrosine residue is present in the peptide. Furthermore, peptide TSTIMTDYNPNYC(#)FAGK shows that tandem mass spectrometry can even distinguish between the various amino acids that can be potentially phosphorylated within a peptide and pinpoint to only one of them as being phosphorylated.

**Figure 5:**
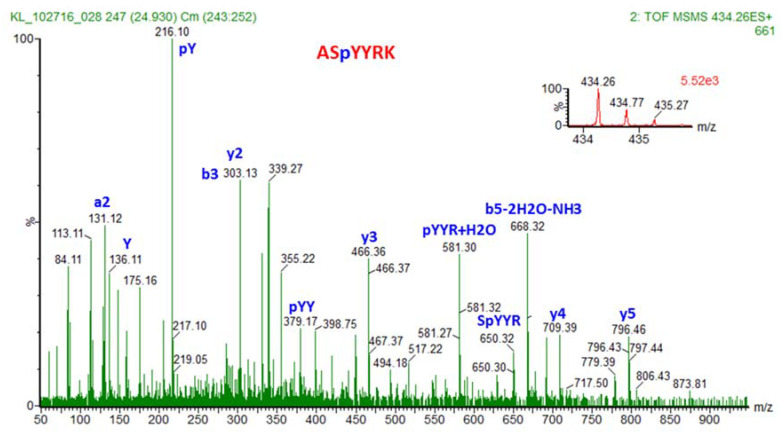
MS spectrum of the precursor ion with *m/z* of 434.26 (2+), shown in the inbox figure, which corresponds to phosphorylated peptide ASpYYRK. Note that pY corresponds to phosphorylated tyrosine residue. Fragmentation of the precursor ion in MS/MS produced a series of fragment ions that correspond to the phosphorylated (bottom) peptide. From three potential amino acids that are phosphorylated (one serine and two tyrosine residues, all neighbors within the peptide sequence ASYYRK, only one tyrosine is phosphorylated, and not the second tyrosine, not the serine residues. This is clearly demonstrated by y3 ion, where tyrosine from the sequence YRK is not phosphorylated, y4 ion, where the second tyrosine from the sequence YYRK is phosphorylated (pYYRK) and y5 ion, where serine residue is not phosphorylated. The *m/z* of the precursor ion indicated that there is only one phosphate group on this peptide ASYYRK (namely ASpYYRK). Therefore, tandem mass spectrometry is highly specific in locating a specific modification within a peptide of a particular amino acid sequence. Reproduced from Reference [205].

An even stronger example is a peptide in which there are three potential phosphorylation sites (SYY), yet the tandem MS/MS is still able to pinpoint with high specificity the amino acid that is phosphorylated and the amino acids that are not (Figure 5). 

### 3.7. MS/MS Applications for Assessment of Quality of Biological Samples and Optimization of Analytical Methods

From a technical point of view, LC-MS/MS studies are useful to assess the quality of biological samples used in proteomic studies. Thereby, the protein expression profiles from flash-frozen samples lysed via homogenization (FF/HOM) that comprise of multiple cell types and optimal cutting temperature-embedded specimens that can undergo laser microdissection (OCT/LMD) to capture and analyze specific tumor and stromal cell types separately from the cell mixture are different, concluding that these two methods cannot be directly compared [206]. Additionally, the LC-MS/MS analysis showed that the initial amount of protein extracted from long-term storage of FFPE tissues is significantly dependent on paraffin block age, the newer blocks yielding more proteins, without an extensive impact on subsequent label-free LC-MS/MS analysis results [207]. A sensitive LC-MS/MS proteomic analysis is useful for the assessment of quality, purity, biochemical enrichment, and recovery of subcellular fractionation of tissue homogenate that provides enriched models, such as nuclei [208]; mitochondria [209]; isolated rough and smooth ER (also studied by MALDI-TOF MS, MALDI-TOF MS/MS, nano-LC-MS/MS, and other proteomic approaches [210]); lysosomes [211,212]; peroxisomes [213]; cytoskeleton [214]; microsomal and cytosolic [215]; exosome-containing [216]; and pure membrane [217] fractions by identifying of protein biomarkers of various organelles [119]. The updates of the in-gel digestion method for protein analysis were performed using LC-MS/MS analysis [59]. LC-MS/MS was also used to identify and assess the efficiency of optimal protocols for in-solution protein digestion [218].

### 3.8. Applications of Tandem MS in In Vitro Studies

LC-MS/MS-based methods allowed the proteomic characterization of an in vitro prostate cancer (CaP) model system, the clonal Prostatic Human Epithelial Cancer (PHEC) cell lines [219]. 2D-LC-MS/MS with tandem mass tagging (TMT) approach was applied to explore the proteomic profile of cellular models of epithelial ovarian cancer (EOC) [220]. LC coupled to tandem mass spectrometry and MALDI–TOF indicated that the representative mitochondrial proteins were associated with glioblastoma in T98G and U87MG cell lines used as glioblastoma models [3]. Additionally, LC-MS/MS-based proteomics may contribute to understanding the development of drug resistance, for example by detection of the overexpression of ABC transporters in resistant cell lines [221]. Many proteins in the mammalian cell culture supernatant containing fetal bovine serum were discovered by the development of a simple albumin depletion approach coupled with DIA-mode LC-MS/MS, with application in the analysis of proteins in conditioned media of HeLa cells with and without tumor necrosis factor stimulation [36]. A shotgun proteomics analysis based on a 2D-LC-MS/MS strategy on a linear ion trap (LQT) mass spectrometer using nano-ESI source in DDA mode demonstrated that the conditioned media of three BC cell lines, as opposed to the cell lysates lead to a significant enrichment in secreted proteins that are potentially useful to be investigated as circulating serum biomarkers in BC [37]. HPLC-MS/MS identified the functional interactome of gastric cancer cell lines (AGS and SGC-7901) that express the HpaA protein which is a surface-localized protein of the gastric pathogen *Helicobacter pylori*, playing a significant role in adhesion to the human gastric epithelial cells, being targeted by antiadhesion drugs against *H. pylori* [222].

### 3.9. Applications of MS/MS in Microbiology and Metaproteomics

MS techniques assure large-molecule analysis for bacterial identification after culture, also offering a high potential for rapid diagnosis of infectious diseases without culture [7]. MALDI-TOF-MS was satisfactory in genus identification of clinical anaerobic bacteria [9]. For rapid identification and antimicrobial susceptibility testing of pathogens, especially Gram-negative bacteria that might be identified at the species level with high accuracy allowing a fast and precise treatment in urinary tract infections, a MALDI-TOF-MS combined protocol has been successfully applied [10]. However, MS-based microbiological testing for blood stream infection based on MALDI-TOF MS has limited resolving power, compared with LC-MS/MS that can identify several thousand peptide sequences, allowing a rapid bacterial identification [65]. A targeted metaproteomic LC-MS/MS analysis was used to assess the diversity of urease produced by ureolytic bacteria in the rumen of cattle; this enzyme catalyzes the hydrolysis of urea into ammonia that can be synthetized into microbial proteins, which support animal growth, as well as milk production [223]. The differentially expressed cell-surface proteins and their abundance quantified by LC-MS/MS in pathogenic *Helicobacter pylori* strains as determinant factors in the etiology of duodenal ulcer, gastric cancer, and autoimmune atrophic gastritis [224] showed differences in their virulence that could be useful for assessing the pathogenicity of these bacteria [225]. 

LC-MS/MS is involved in the elucidation of interactions between viruses, such as HIV-1, and the host cells, by proteomic organelle mapping [226]. Quantitative isobaric tags for relative and absolute quantification (iTRAQ)-LC-MS/MS proteomics revealed the upregulated and downregulated proteins of DF-1 cells after infection with an avian oncogenic virus that induces tumors and causes severe economic losses, emphasizing the viral pathogenic mechanisms and virus-host interactions [227]. A new specific and rapid method for SARS-CoV-2 identification is based on six peptide markers identified by LC-MS/MS as a complementary assay to the standard RT-qPCR method of detection [228]. 

### 3.10. Applications of MS/MS in Foodomics

LC-MS/MS was used for identification and characterization of the bovine milk proteome under normal and pathological conditions, such as coliform mastitis [229] or subclinical mastitis by coagulase-negative staphylococci, where LC-ESI-MS/MS revealed significant changes in the cow milk peptidome, emphasizing putative biomarkers and detecting consequences for characteristics of cow milk and dairy products [230]. Additionally, LC-ESI-MS/MS analysis identified many peptides that belong to phage proteins from a mastitis-causing strain *Streptococcus* spp. isolated from milk [231].

LC-MS/MS-based assays may be developed for detecting fish fraud at the protein level to assure the authenticity of seafood products [232]. This is also one of the main proteomics approach for detection and quantification of seafood allergens known as proteins with IgE reactivity [233]. A nontargeted proteomic-based method using an RP-LC system coupled with a highly sensitive QTOF hybrid mass spectrometer by a DuoSpray Ion Source was performed in DDA and DIA acquisition modes to discover putative candidate peptide biomarkers able to discriminate Canadian farmed from wild type salmon muscle samples [76]. For meat-based food authentication and fraud detection, HPLC-MS/MS detected undeclared caseins and whey proteins in meat products that can be a problem for persons suffering from allergies [234], as well as legume proteins in various emulsion-type sausages [235]. Infusion MS/MS analysis enabled discrimination between horse meat, beef and pork proteins in cooked and smoked sausages [236]. 

Due to its potential terrorist use, the detection of the protein toxic ricin in crude toxin preparation is necessary by MALDI-MS, the purified ricin being characterized by LC-ES MS/MS [237]. Modern approaches in the identification and quantification of immunogenic peptides in cereals were performed by LC-MS/MS, the gluten peptides being involved in celiac disease and gluten-related allergies [238]. 

The functional food based on high concentration of bioactive compounds, such as plant extracts, nutraceuticals, and supplements [239], requires tandem MS-based proteomic strategies to emphasize their proteome/peptidome biomolecular structure and mechanisms involved in improving symptoms in various diseases, such as prevention of myocardial infarction by intake of an extract from common wheat grain [240] or amelioration of metabolic syndrome via the administration of various types of tea types aqueous extracts with effects on the liver proteome [241]. MALDI-TOF-MS and HPLC-ESI-MS/MS approaches contributed to a better understanding of the influence of different processing methods, such as post-harvest treatment prior to roasting, on protein quality in green coffee beans, correlated with coffee cup quality and aroma [242], as well as for the identification of cyclic dipeptides, known as taste compounds that occur in different foods, like the tea extract [243]. Proteins identified in ginseng root and cauline leaf, respectively, provided a better understanding of ginsenoside synthesis and guidance for artificial cultivation [244].

### 3.11. Applications of Tandem Mass Spectrometry in Exposomics and Environmental Pollution

LC-MS/MS helps to detect trace substances in environmental samples [245], as well as to explore the impact of the environmental pollutants, such as microplastics and heavy metals, on proteome-level activity in antibiotic-resistant bacteria in the environment [246]. Exposomics studies based on non-target protein analysis of samples from wastewater treatment plants used LC coupled with high-resolution tandem mass spectrometry with modern soft ionization techniques [78]. In the exposomics field, LC-MS/MS analysis of skin biopsy samples can be used to assess the proteomic landscape after in vivo exposure to the irritant agents [247].

## 4. Conclusions

In conclusion, in this work, we reviewed the basic principles of tandem mass spectrometry methodology, including sample preparation, MS/MS detection, and applications in biomedicine. Tandem mass spectrometry methods can be applied in any field of biological and both fundamental and clinical research for qualitative and quantitative bioanalysis of proteins and peptides, as well as for their PTMs and protein–protein interactions (PPIs), in any normal or pathological biological sample.

Large-scale implementation of laboratory automated LC-MS/MS instruments and applications of MS in clinical settings can open a window for the establishment of faster, easier, and accurate clinical diagnostic decisions in personalized, preventive, and “point of care” medicine. The development of alternative testing methods based on tandem MS techniques offers a higher precision compared with the traditional/conventional assays. Additionally, advances in MS/MS techniques allow the transition from targeted to untargeted proteomic approaches, sustaining the development of new omics fields, such as PTM-omics and PPI-omics, with a high impact for a better understanding of the normal or pathological states of cells and disease mechanisms. LC-MS/MS can be used as a complementary assay to the RT-qPCR standard method for the identification of new viruses. Newborn screening, fetal diagnostics based on proteomics, clinical endocrinology, the identification of new proteins and peptides as bioactive compounds in functional food, pharmacokinetics studies, biological control of pests, or the detection of protein alterations associated with age and age-related diseases can also benefit from recent and future advancements in tandem mass spectrometry-based methods.
